# The Determinants of Leptin Levels in Diabetic and Nondiabetic Saudi Males

**DOI:** 10.1155/2017/3506871

**Published:** 2017-03-01

**Authors:** Mona Hmoud Al Sheikh

**Affiliations:** Department of Physiology, College of Medicine, University of Dammam, Dammam, Saudi Arabia

## Abstract

*Objective.* This study aimed to identify the main determinants of serum leptin levels. *Methods.* A sample of 113 Saudi adult males (55 diabetic and 58 nondiabetic) was selected according to the inclusion and exclusion criteria identified below. Blood samples were taken from participants after fasting for 12 hours. For diabetic patients, the insulin dose was given 12 hours before. In general, the study instrument consisted of blood biochemical tests. Metabolic parameters, glycosylated hemoglobin (HbA1c), low-density lipoprotein (LDL), high-density lipoprotein (HDL), cholesterol, and triglyceride (TG), and adipokines, leptin, adiponectin, visfatin, and resistin, were measured. Multivariate model was utilized to identify the relationship between leptin levels and the independent variables. *Results.* When adjusted for resistin in the diabetic group, the results demonstrated a significant relationship between visfatin, LDL and TG, and leptin levels (*p* < 0.05). However, when controlled for resistin, the effect of LDL and TG disappeared while that of visfatin stayed in the model. For the nondiabetic group, the results indicated a significant relationship between insulin, BMI, and leptin levels when adjusted for resistin (*p* < 0.05). However, the effect of insulin disappeared when the model was controlled for resistin. The study results found no relationship between leptin and adiponectin levels in either the diabetic or nondiabetic group and whether adjusted or controlled for resistin. *Conclusion.* This study provided better understanding of the metabolism of leptin and unveiled the major determinants of leptin levels in diabetic and nondiabetic males. In conclusion, these results show that the association between leptin and metabolic parameters decreases with the progress of disease.

## 1. Introduction

The prevalence of obesity has dramatically increased in the past ten years among the world's population [1]. According to Blüher and Mantzoros [2], obesity has increased the incidence of diet-related diseases such as type 2 diabetes, hypertension, and cardiovascular diseases.

The common features of these diseases are eating and overfeeding which consequently lead to obesity and disease [3–7]. Biologically, researchers have attributed that to adipose tissue which is regarded not only as an energy-storing organ but also as an endocrine organ [8]. It releases a number of humoral factors known as adipokines [9]. It is a group of endocrine organs and can be divided into white adipose tissue and brown adipose tissue [10]. Visceral and subcutaneous adipose tissues are known to be the most abundant sites of adipose tissues in the body. Such tissues produce adipokines [11]. It plays an important role in insulin resistance through production of adipose-derived proteins. The adipokine molecules belong to numerous functional categories including endocrine function that are related to leptin, adiponectin, and visfatin. Furthermore, adipokines play a major role in a number of metabolic functions as well as in the control of energy metabolism [12].

Leptin is produced from the ob gene [13] that contributes to body weight regulation. Leptin plays a critical role in human pathophysiology of a group of diseases because it elicits considerable interest in its potential in treating obesity and insulin resistance [14]. In other words, evidence suggests that leptin is a protein involved in the pathology of obesity [15]. Empirical studies suggest that serum leptin is critical in regulating blood sugar through two different brain passage ways. The first one is responsible for controlling appetite and fat while the second one is responsible for telling the liver what to do with its stored glucose [16]. According to Tuominen et al. [17], there is a positive interaction between insulin and leptin. Additionally, leptin is linked with body fat percentage, BMI, and insulin concentration. Leptin resistance entails different interpretations since its complexity gives a range of definitions. Resistance may occur due to inability of serum leptin to reach or influence target sites within the brain [18].

Adiponectin is another adipokine released by adipose tissue. According to a number of studies, adiponectin concentration decreases in obese people and patients with type two diabetes and hypertension [19]. Furthermore, adiponectin decreases plasma triglycerides by decreasing cholesterol levels in the blood. According to Jurimae et al. [20], adiponectin plays a major role in glucose and lipid metabolism. Therefore, it is thought to enhance insulin resistance as well as reduce very-low-density lipoprotein (VLDL) cholesterol which decreases the incidence of dyslipidaemia [21].

Visfatin is another insulin-resistance-enhancing factor which is determined by obesity and type 2 diabetes. It was basically defined by Samal et al. [22] as “a novel human pre-B-cell colony-enhancing factor (PBEF).” Visfatin consists of ten introns and eleven exons that are spanned over 34.4 Kb of genomic DNA. Visfatin is a unique adipokine that is mainly secreted by visceral adipose tissue [23]. According to a number of studies, visfatin levels are increased in type two diabetes patients and it is associated with insulin resistance [23–26]. Although visfatin is related to insulin secretion, no studies have shown an association between visfatin and insulin sensitivity.

Resistin is another adipokine which is regarded as a derived signalling cysteine-rich molecule and made of 144 amino acids. It is involved in different processes of inflammation. Additionally, human resistin has been detected in various tissues such as the stomach, thymus, placenta, thyroid gland, and skeletal muscle [27]. Clearly from its name, resistin *resists* the action of insulin and predominantly increases in obese people. This leads to a proinflammatory molecule that plays a critical role in the pathogenesis of diabetes and its complications [28]. Furthermore, researchers suggested that the hormone resistin links between diabetes and obesity [29].

On the basis of this brief description of adipose tissues and their role in insulin resistance, this study aims to determine the main factors that contribute to change in the leptin levels in diabetic and nondiabetic Saudi males. It is worth emphasizing that there are only a few studies that have addressed the leptin levels in males in general and Saudi males in particular. The majority of studies have investigated leptin in females in relation to diabetes and obesity. Therefore, this study is distinguished by its originality and findings. The study assumed positive relationships between leptin levels (dependent variables) and the independent variables or otherwise.

## 2. Materials and Methods

### 2.1. Sample

The sample of the study consisted of 113 Saudi males (55 diabetic and 58 nondiabetic) who were selected according to certain inclusion and exclusion criteria. Inclusion criteria for both groups were comprised of absence of chronic diseases, for example, cardiac diseases, hypertension, and hyperlipidaemia. Furthermore, participants were nonsmokers and had no infectious diseases. For diabetic patients, the inclusion criteria were diagnosis of diabetes for more than 5 years.

All the study procedures were approved by the Ethics Committee of Biological and Medical Research for the University of Dammam (HAP-05-D-003). All the participants were asked to sign the informed consent form provided.

### 2.2. Blood Samples

The study assured that participants were fasting at least 12 hours. Blood samples were collected with the help of blood bank endocrine unit at King Fahd Hospital of the university. The serum was kept at temperature of −68°C.

For diabetic participants, the last dose of insulin was 12 hours prior to sample withdrawal (all patients received regular insulin and neutral protamine Hagedorn (NPH) with an average total dose of 0.84/kg/day range of 0.6–1.0). The study tools included blood biochemical tests, collected in nonheparinized test tubes, then centrifuged and kept at 18°C in a deep freezer.

### 2.3. Metabolic Parameters

Fasting serum glucose, HbA1c, LDL, HDL, cholesterol, and TG were measured using SIEMENS stream lab-dimension clinical chemistry system-RxL, Max (USA) kit. Inclusion was measured in serum using Abbott AxSYM system produced by Axis-Shield Diagnostics Ltd., UK. The AxSYM inclusion assay is based on the microparticle enzyme immunoassay (MEIA) technology.

### 2.4. Adipocytokine Assays

Leptin, adiponectin, visfatin, and resistin were measured using ELISA, Phoenix Pharmaceuticals, USA.

### 2.5. Statistical Analysis

Data were analyzed using SPSS Base 22. Descriptive statistics analysis was used for characteristics including leptin levels, BMI, visfatin, insulin, resistin, and adiponectin which were used later as independent (predictors) variables. A *p* value less than 0.05 was regarded to be statistically significant.

In relation to inferential statistics, multiple regression analysis was used to determine the main factors affecting serum leptin levels before and after adjustment for TG. Serum leptin level was used as the dependent variable along with several independent variables. Multiple regression analysis was run twice for diabetic and nondiabetic subjects which produced four models (two for diabetic and two for nondiabetic). For the diabetic male patients, two models were constructed where the first one was adjusted for resistin while the second model was controlled (not adjusted) for resistin. The same procedure was performed for the nondiabetic group. Resistin, as an independent variable, was chosen to be adjusted and controlled for in the regression models. This technique was used to reveal the impact of resistin on the action of insulin. As mentioned above, resistin plays an important role in the pathogenesis of diabetes and its complications [8].

Because the data is continuous and normal, it was beneficial to use multiple regression analysis. In order to transform the leptin results into normal distribution, log transformation was used. The presignificance level *α* = 0.05 was used to measure the strength of the relationship between the leptin levels and the independent variables (factors). The four multiple regression models were constructed. The *first* model targeted diabetic participants adjusted for TG and other variables while the *second* model has not included TG. The *third* model targeted nondiabetic participants and adjusted for TG and other independent variables while the *fourth* model adjusted for all independent variables but without TG.

## 3. Results

### 3.1. Descriptive Analysis

Background characteristics of metabolism of the diabetic and nondiabetic subjects are shown in Table 1. We found no significant difference between the diabetic and nondiabetic subjects in relation to BMI (29.559 kg and 27.764 kg, resp.). There was significant difference in HbA1c between the diabetic (8.59) and nondiabetic (5.57) subjects. However, cholesterol did not show significant difference between the 2 groups (173.99 versus 183.23). There were also no significant differences between diabetic and nondiabetic males in terms of HDL (48.548 and 42.590, resp.). As expected, the average of LDL is higher in the diabetic (165.9032) than that in the nondiabetic (119.6752). TG average is lower in the diabetic (167.2901) than that in the nondiabetic (194.0725). Serum leptin levels were moderately higher in the diabetic (58.0671) than those in the nondiabetic (50.1984). In relation to adiponectin, the results show no difference between the two groups (10.8007 and 10.2038, resp.). Visfatin is higher in diabetics. However, insulin is lower in the diabetic (32.1458) than that in the nondiabetic (40.8289). There is a slight difference between diabetic (5.7642) and nondiabetic (4.6751) subjects in relation to resistin.

### 3.2. Inferential Analysis: Modelling

#### 3.2.1. Model One: Diabetic Males

*(1) Model of Diabetic Participants Adjusted for Resistin (Table 2). *Model 1 targeted diabetic participants and adjusted for resistin as an independent variable. The model summary represented in *R* square = 0.618 indicates that 61.8% of the total variance in the leptin levels has been explained from the independent variables. The results of ANOVA demonstrate an overall relationship between the leptin levels and independent variables. The results in the coefficient table demonstrate significant relationship between the leptin levels and LDL (*p* = 0.044) and visfatin (*p* = 0.000). However, multiple regression analysis indicated no significant relationship between leptin levels and adiponectin (*p* = 0.171), insulin (*p* = 0.620), BMI = 0.842, and resistin (*p* = 0.166).

*(2) Model of Diabetic Participants Controlled for Resistin (Table 2).* This model was constructed without adjustment for resistin. Firstly, 75.9% of the total variation in the dependent variable (leptin) was explained (*R* square = 0.759) from the independent variables. The results of ANOVA demonstrate an overall relationship between the leptin levels and independent variables (*F* = 4.536, *p* = 0.005). It is clear from the coefficients table that the effect of LDL and TG on the leptin level disappeared (*p* = 0.093) when controlled for resistin while other factors such as visfatin, insulin, adiponectin, and BMI had no significant association with leptin (*p* = 0.842).

#### 3.2.2. Model Two: Nondiabetic Males

The results in Table 3 demonstrate a significant relationship between leptin levels and insulin as well as BMI (*p* = 0.048, 0.009, resp.) when adjusted for resistin. When the variable resistin was controlled for (removed from the multiple regression model), the relationship between leptin levels and insulin disappeared (*p* = 0.061) while staying significant in relation to BMI (*p* = 0.005). It is clear from Table 3 that there is no significant relationship between leptin levels and other independent variables examined in the regression model such as LDL, adiponectin, visfatin, and TG.

## 4. Discussion and Conclusions

The literature documents that since the discovery of serum leptin as a messenger secreted by adipose tissue, its role in the endocrinology field has increased tremendously. For this reason, studying different sections of the population is imperative in addressing the factors affecting the levels of leptin in diabetic patients. To our knowledge, this is the first study conducted on Saudi males that examined the leptin levels in diabetics and nondiabetics taking into account a number of factors. In this study, the author sought to determine the relationship between leptin level and other adipokines and metabolic parameters in male subjects.

### 4.1. Relationship between Leptin and Diabetes

The study results indicated low levels of leptin in both the diabetic and nondiabetic subjects (58 and 50, resp.). These results suggest that low leptin levels may be attributed to insignificant relationship between BMI and leptin in diabetic male patients (*p* = 0.842) and significant relationship between BMI and leptin in nondiabetic males (*p* = 0.009). Abu-Sayeed et al. [30] found that lean diabetic patients have shown low leptin levels. These results were also reported by many other researchers [14, 31, 32]. Haffner et al. [33] found no differences in the concentration of leptin levels between diabetic and nondiabetic subjects as well as no differences between obese and nonobese subjects. Results of different studies conducted on diabetes and leptin have been inconsistent. For instance, some studies demonstrated an association between diabetes and leptin levels [33–35] while other studies found no significant relationship [36, 37]. However, some other studies indicated inverse correlation between the two variables [38–40].

### 4.2. Relationship between Leptin Levels and BMI

The results of multiple regression demonstrated no significant relationship between BMI and leptin levels in diabetic males (*p* = 0.842) while there was a significant association between the two variables in nondiabetic males (*p* = 0.009). In relation to this aspect, Bandaru and Shanker [41] found an association between diabetes and leptin when BMI was adjusted in the regression model. Ahren et al. [42] stressed that leptin levels are increased in patients with high BMI levels (obese). However, other studies argued that leptin levels were reduced in obese and diabetic patients, which attributed to the altered fat distribution [14, 32]. Matsubara et al. [43] found that the concentration of serum leptin in women with high levels of BMI was increased compared to those in the middle and/or lower BMI group.

With regard to nondiabetic subjects, Tatti et al. [14] found a significant relationship between leptin levels and BMI in nondiabetic women but not in nondiabetic men. The authors attributed the gender differences to the small sample of male participants. Esteghamati et al. [44] found no significant difference in leptin levels between diabetic and nondiabetic subjects when adjusted for BMI.

### 4.3. Relationship between Leptin Levels and Adiponectin

The results of multiple regression demonstrated no significant relationship between leptin levels and adiponectin in both the diabetic and nondiabetic subjects (*p* = 0.171) with and without adjustment for resistin. These results come in line with other studies which found a negative and inverse relationship between leptin and adiponectin in nondiabetic subjects [43, 45]. Nevertheless, Smith et al. [46] found significant relationship between leptin and adiponectin. Weyer et al. [47] found that adiponectin was decreased in patients who had insulin resistance while Arita et al. [48] indicated that adiponectin was decreased in patients with obesity. It is believed that leptin increases with obesity in patients with diabetes [42] while adiponectin decreases in obese patients with diabetes.

Ahren et al. [42] and Ebihara et al. [49] indicated that adiponectin was negatively correlated with serum leptin concentration and fasting insulin as well as insulin resistance. In humans, leptin and adiponectin levels are strongly associated in the obese [15, 46].

### 4.4. Relationship between Leptin Levels and LDL

The results of multiple regression analysis showed significant relationship between leptin levels and LDL in diabetic subjects (see Table 2, *p* = 0.044) after adjustment for resistin while this significant relationship disappeared when resistin was removed from the model. This result suggests that leptin levels are not correlated with increases in serum resistin level. Hiroso et al. [50] in their study on Japanese subjects showed that there was a significant relationship between LDL and serum leptin levels. These results are consistent with the study conducted by Njajou et al. [51] who found a positive and significant relationship between leptin and LDL in white subjects but no significant relationship in black subjects. However, the results of multiple regression showed no significant relationship between leptin levels and LDL in nondiabetic subjects (see Table 3, *p* = 0.129). In conclusion, LDL is significantly correlated with the leptin levels after adjustment for resistin. This result suggests that the leptin levels are correlated with increases in the level of resistin.

### 4.5. Relationship between Leptin Levels and Visfatin

Multiple regression analysis demonstrates significant relationship between leptin levels and visfatin (*p* = 0.000) in diabetic males while showing no significant relationship in nondiabetic males (*p* = 0.348, 0.343). According to Adeghate [52], visfatin is an adipocyte hormone that has a direct relationship with diabetes mellitus. The author argues that visfatin binds to insulin receptor and causes hypoglycaemia through the reduction of blood glucose by stimulating its utilization in adipocytes and myocytes as well as reducing its release from liver cells. Fukuhara et al. [23] found that visfatin has an insulin mimetic effect which includes inhibiting hepatic glucose release and augmentation of glucose uptake in adipocytes. Additionally, the authors conclude that visfatin increases triglyceride synthesis and its accumulation in preadipocytes. Nonetheless, according to Esteghamati et al. [44], diabetic patients were found to have higher visfatin and leptin levels while having lower adiponectin levels compared to nondiabetic subjects.

### 4.6. Relationship between Leptin Levels and TG

The study results showed a significant relationship between TG and leptin levels in diabetic patients (Table 2, *p* = 0.047) after adjustment for resistin while this significant relationship disappeared when resistin was taken out from the multiple regression model (*p* = 0.958). Regarding nondiabetic participants, the results demonstrated an insignificant relationship between leptin levels and TG. This was due to the weak and negative correlation between resistin and TG (*r* = −0.104). Banks et al. [53] found that serum triglycerides are elevated in both starvation and obesity which inhibited leptin transportation. This was due to the fat component of milk (98% triglycerides) which induces inhibition of leptin transport across the brain in vivo, in situ, and in vitro. The authors stressed that triglycerides directly caused leptin resistance [53].

### 4.7. Relationship between Leptin Levels and Insulin

The study results indicated a positive relationship between leptin and insulin in nondiabetic males (*p* = 0.048) after adjustment for resistin. This result is consistent with the study results conducted by Lichnovsak et al. [54] who found a significant correlation between leptin and insulin. Lichnovsak et al. [54] suggest that the positive and significant relationship between leptin and insulin increases insulin resistance and its parameters such as proinsulin and mainly C-peptide. In relation to diabetics, the multiple regression model showed no significant relationship between serum leptin and insulin. Patrizio et al.'s [55] study demonstrated an association between insulin and leptin concentration, but the statistical significance was borderline. According to Pelleymounter et al. [56], changes in blood glucose levels come before the changes in body weight which suggests that leptin may have a direct effect on insulin action and increases insulin resistance. Marchesini et al. [57] suggested that insulin resistance impairs fatty acid oxidation which exports from the liver in the form of very-low-density lipoprotein and promotes de novo lipogenesis.

### 4.8. Conclusions of the Paper

This study provided better understanding of lipid and glucose metabolism and unveiled the major factors affecting the leptin levels in diabetic and nondiabetic males. In conclusion, these results show that the association between leptin and metabolic parameters decreases with the progress of disease. Briefly, the study found that visfatin, LDL, and TG were the main factors associated with the leptin levels in Saudi male diabetic patients while BMI and insulin were the major factors correlated with leptin levels in male nondiabetic subjects.

## Figures and Tables

**Table 1:** Background characteristics of the subjects in the two groups diabetic and nondiabetic.

Characteristics	Diabetic *n* = 55 Mean (SD)	Nondiabetic *n* = 58 Mean (SD)
BMI	29.559 (6.340)	27.764 (6.028)
HbA1c	8.587 (2.097)	5.572 (0.423)
Chol	173.983 (57.614)	183.230 (40.482)
HDL	48.548 (15.777)	42.590 (10.710)
LDL	165.903 (39.166)	119.675 (32.284)
TG	167.291 (42.962)	194.072 (51.511)
Leptin	58.067 (23.385)	50.298 (17.286)
Adiponectin	10.801 (5.678)	10.204 (3.534)
Visfatin	8.101 (4.427)	5.753 (2.782)
Insulin	32.146 (9.368)	40.829 (6.156)
Resistin	5.764 (2.483)	4.675 (1.596)

**Table 2:** Serum leptin levels in diabetic males (multiple regression model adjusted and controlled for resistin).

Variable	Adjusted for resistin (significance, *p* value)	Controlled for resistin (significance, *p* value)
LDL	0.044	0.093
Adiponectin	0.171	0.165
Visfatin	0.000	0.001
Insulin	0.620	0.847
TG	0.047	0.093
BMI	0.842	0.988
Resistin	0.166	—

**Table 3:** Serum leptin levels in nondiabetic males (multiple regression model when controlled for resistin).

Variable	Adjusted for resistin (significance, *p* value)	Controlled for resistin (significance, *p* value)
LDL	0.129	0.176
Adiponectin	0.084	0.092
Visfatin	0.348	0.343
Insulin	0.048	0.061
TG	0.891	0.958
BMI	0.009	0.005
Resistin	0.277	—

## Abbreviations

ANOVA: Analysis of variance
BMI: Body mass index
ELISA: Enzyme-linked immunosorbent assay
HbA1c: Glycosylated hemoglobin
HDL: High-density lipoprotein
LDL: Low-density lipoprotein
PBEF: Pre-B-cell colony-enhancing factor
TG: Triglyceride
VLDL: Very-low-density lipoprotein.
